# Endophytic Bacteria *Enterobacter hormaechei* Fabricated Silver Nanoparticles and Their Antimicrobial Activity

**DOI:** 10.3390/pharmaceutics13040511

**Published:** 2021-04-08

**Authors:** Tahmina Monowar, Md. Sayedur Rahman, Subhash J. Bhore, Kathiresan V. Sathasivam

**Affiliations:** 1 Unit of Microbiology, Faculty of Medicine, AIMST University, Bedong 08100, Kedah, Malaysia; 2 Government of the People’s Republic of Bangladesh, Ministry of Information, Bangladesh Betar, Mymensingh 2202, Bangladesh; sayed_radio@yahoo.com; 3 Department of Biotechnology, Faculty of Applied Sciences, AIMST University, Bedong 08100, Kedah, Malaysia; subhashbhore@gmail.com (S.J.B.); kathir.aimst@gmail.com (K.V.S.)

**Keywords:** endophytic bacteria, *Enterobacter hormaechei*, silver nanoparticles, pathogenic microbes, multidrug resistant bacteria

## Abstract

Antimicrobial resistance (AMR), one of the greatest issues for humankind, draws special attention to the scientists formulating new drugs to prevent it. Great emphasis on the biological synthesis of silver nanoparticles (AgNPs) for utilization in single or combinatorial therapy will open up new avenues to the discovery of new antimicrobial drugs. The purpose of this study was to synthesize AgNPs following a green approach by using an endophytic bacterial strain, *Enterobacter hormaechei*, and to assess their antimicrobial potential against five pathogenic and four multidrug-resistant (MDR) microbes. UV-Vis spectroscopy, fourier-transform infrared spectroscopy (FTIR), transmission electron microscopy (TEM), scanning electron microscopy-energy dispersive X-ray spectroscopy (SEM-EDX), and zeta potential (ζ) were used to characterize the synthesized AgNPs. Endophytic *E. hormaechei*-mediated AgNPs (Eh-AgNPs) were represented by a strong UV-Vis absorbance peak at 418 nm within 5 min, forming spherical and polydispersed nanoparticles in the size range of 9.91 nm to 92.54 nm. The Eh-AgNPs were moderately stable with a mean ζ value of −19.73 ± 3.94 mV. The presence of amine, amide, and hydroxyl functional groups was observed from FTIR analysis. In comparison to conventional antibiotics, the Eh-AgNPs were more effective against *Bacillus cereus* (ATCC 10876) and *Candida albicans* (ATCC 10231), exhibiting 9.14 ± 0.05 mm and 8.24 ± 0.05 mm zones of inhibition (ZOIs), respectively, while displaying effective inhibitory activity with ZOIs ranging from 10.98 ± 0.08 to 13.20 ± 0.07 mm against the MDR bacteria. Eh-AgNP synthesis was rapid and eco-friendly. The results showed that Eh-AgNPs are promising antimicrobial agents that can be used in the development and formulation of new drugs to curb the menace of antimicrobial resistance in pathogenic and MDR microbes.

## 1. Introduction

Antimicrobial resistance (AMR) is a state where microorganisms become resistant to antimicrobial drugs, resulting in ineffective treatment with existing antibiotics. AMR is a global crisis that poses a terrifying challenge to the achievement of universal health coverage. Alarming levels of resistance jeopardize the advancement of many sustainable development goals, including health. The misuse and overuse of persistent antimicrobials have heightened the development and extent of AMR. Increases in AMR are driven by the spread of microbes exposed to antimicrobial compounds and their mechanisms of resistance. AMR is accelerated when antimicrobial compounds are persistent in the environment or in the microbes’ hosts [1]. Presently, about 0.7 million deaths occur annually worldwide owing to drug-resistant diseases [2]. Without a sustained effort to control AMR, mortality arising from drug-resistant diseases could rise to 10 million globally per year by 2050 in the worst-case scenario [2]. Nonetheless, AMR is a growing crisis worldwide, causing 25,000 deaths in the European Union (EU), over 58,000 infant deaths in India, over 38,000 deaths in Thailand, and over 23,000 deaths in the United States per year [3]. In fact, the whole world is now experiencing the globalization of AMR. If the present trend continues without suitable strategies, it will impart an appalling human and economic cost of about 10 million deaths every year by 2050, with an estimated reduction of nearly 3.5% in gross domestic product (GDP). Moreover, the consequences of these drug-resistant infections would cost the world nearly US$100 trillion [4]. Presently, the 32 available antibiotics in the clinical pipeline targeting priority pathogens are insufficient to challenge the problem of extensive or pan-drug-resistant Gram-negative bacteria [5]. In addition to antimicrobial stewardship programmes, the formulation of new antibacterial compounds is warranted to ensure continual effective treatment of drug-resistant bacterial infections [5].

Nanotechnology, an evolving field of biomedical research, possesses tremendous promise as a tool for the development of alternative and novel antimicrobial agents [6]. Recent reports in the literature suggest that silver nanoparticles (AgNPs) possess antibacterial potential against a diverse range of pathogenic microbes as well as drug-resistant pathogens [7,8,9,10,11,12,13,14]. Therefore, scientific efforts to synthesize tailor-made AgNPs in different sizes and shapes and evaluate their antimicrobial efficiencies have gained momentum in recent decades [8,12].

Out of the four different (i.e., photochemical, biological, physical, and chemical) methods of synthesizing AgNPs, the biological method is regarded as easy, safe, eco-friendly, and economical and is being extensively studied [12,13,14,15,16,17]. Biologically synthesized AgNPs are better in comparison to chemically synthesized AgNPs in terms of their antimicrobial properties [14]. The production method is a greener concept in which silver atoms are congregated to form nanomaterials in sizes ranging from 1 to 100 nm by the bottom-up technology [12]. Different types of bacteria, fungi, yeasts, plants, seaweeds, algae, etc. are being used in this biological approach [12,13,15,16,17]. Recent trends show that AgNP synthesis using bacteria is attractive, cost-effective, and easy; these factors have led to an increased number of reports on AgNP synthesis using different Gram-positive and Gram-negative genera and species [9,13]. Nanoparticle studies with plant-derived materials are the most predominant, accounting for 40%, followed by bacteria, biomolecules, fungi, and algae at 34%, 15%, 7% and 4%, respectively [12]. Different biomolecules such as phenolics, flavonoids, proteins, amino acids, carbohydrates, vitamins, alcoholic compounds, carboxylic acids, etc. that are present in biological samples are assumed to act as capping, reducing, and stabilizing agents in the synthesis of AgNPs [13,16]. However, an increased number of bacteria for use in the synthesis of AgNPs would be advantageous in combating pathogenic bacteria. This would also open novel avenues for their application [9].

Endophytes are microorganisms that inhabit the tissues of living plants and possess a resourceful reservoir of secondary metabolites with therapeutic potential [18]. Endophytic bacteria are a good source of biomaterials for the synthesis of AgNPs that could be utilized as effective antimicrobial agents [19,20,21,22,23]. The sustainable and large-scale production of AgNPs with cost-effective and eco-friendly methods is one of the major advantages of bacteria-mediated synthesis of AgNPs [14,15,16,17]. Moreover, being less time-consuming, extracellular synthesis is more advantageous than the intracellular method [14]. Extracellular synthesis involves diffusion of metal ions through cell membranes, where the metal ions are reduced to metal nanoparticles by cytoplasmic redox mediators [12].

*Enterobacter hormaechei* is a Gram-positive opportunistic bacterium found in animals [24] and humans that causes clinical nosocomial infections [25,26]. Recently, drug-resistant strains of this species have been reported in different parts of the world [26,27]. Under the present global scenario of antimicrobial resistance, more emphasis has been placed on innovation and research to discover, exploit, apply, and assess novel therapies for antibiotic-resistant infections through the identification of alternative treatments and prevention solutions [1]. Endophytic microbes produce a large variety of secondary metabolites of therapeutic importance that have made them an important agent for drug discovery [18]. Furthermore, emphasis has been placed on the exploration of endophytes as a reliable storehouse of bioactive and chemically novel compounds that may be used in combating numerous pathogenic and drug-resistant microbes affecting humans [18]. Recent reports in the literature suggest that AgNPs fabricated with endophytic bacteria possess effective antimicrobial activities [23,28,29,30,31]. Taking into account the bio-prospects of endophytic bacteria, the present study aimed to explore the biosynthesis of AgNPs using endophytic *E*. *hormaechei* and characterize the synthesized bacteria and their possible applications as antimicrobial agents against pathogenic microbes and multidrug-resistant (MDR) bacteria.

## 2. Materials and Methods

### 2.1. Culture of the Endophyte and Preparation of Cell-Free Extract

Isolates of the endophytic *Enterobacter hormaechei* (GenBank accession no HQ694367), which had been previously assessed for molecular identification using 16S rRNA [32], were collected from the laboratory of AIMST University, Malaysia. The isolates were grown on nutrient broth (HiMedia Lab. Ltd., Mumbai, India) at 37 °C for 24 h, then taken into 250 mL sterile Luria Bertani broth (HiMedia Lab. Ltd., Mumbai, India) and incubated (37 °C, 24 h, 180 rpm) in a rotary incubator shaker (Innova 40, New Brunswick Scientific Co., New York, NY, USA). The overnight culture was transferred into a 50 mL Beckman tube (Beckman Coulter, Inc., Pasadena, CA, USA) and the bacterial cells were centrifuged at 8000 rpm for 10 min at 4 °C using a centrifuge machine (Avanti J-26 XPI, Beckman Coulter, Inc., Pasadena, CA, USA). The resulting supernatant, termed as cell-free extract (CFE), was used immediately for extracellular synthesis of AgNPs.

### 2.2. Synthesis of Eh-AgNPs

Freshly prepared CFE was added to 100 mL of 0.1 mM silver nitrate (2%, *v*/*v*) solution (AgNO_3_, Fisher Scientific, Hampton, NH, USA) and was exposed to bright sunlight (temperature: 32 °C, solar intensity: ~72,000 lux) for a period of 60 min. The formation of Eh-AgNPs was monitored at regular time intervals (5, 15, 30, and 60 min) through observation of the colour change pattern of the reaction mixture. Simultaneously, a similar experiment was carried out under dark conditions (25 °C, 0 lux, 24 h) to explore the role of sunlight in Eh-AgNP synthesis. AgNO_3_ solution (0.1 mM) without the presence of CFE was used as the negative control. Pellets of the synthesized nanoparticles in the reaction mixture were centrifuged at 12,000 rpm for 15 min at 4 °C using the centrifuge machine. The pellets were washed (×3) with deionized water and dried in a vacuum dryer (Yamato Scientific Co. Ltd., Tokyo, Japan).

### 2.3. Characterization of Eh-AgNPs

UV-Vis spectroscopy, fourier-transform infrared spectroscopy (FTIR) spectroscopy, TEM, SEM-EDX, and Zeta potential were used to characterize the fabricated Eh-AgNPs [23]. The UV-Vis spectrum of the synthesized Eh-AgNPs was recorded in the range of 200–800 nm using a UV-Vis spectrophotometer (DU-800, Beckman Coulter, Inc., Pasadena, CA, USA). FTIR spectroscopy of the Eh-AgNPs was obtained using an FTIR spectrophotometer (PE 1600, GMI Inc., Ramsey, MN, USA) over the spectrum of 400–4000 cm^−1^ at a resolution of 4 cm^−1^. Morphology, size, and distribution of the Eh-AgNPs were observed using a Philips CM 12 TEM system equipped with Philips Docu Version 3.2 image analysis software (Philips Electron Optics, Eindhoven, The Netherlands) at 120 kV. Further morphology of the Eh-AgNPs was observed using scanning electron microscopy (SEM, Phenom-World B.V., Eindhoven, The Netherlands) running at 15 kV. Energy dispersive X-ray (EDX, Phenom-World B.V., Eindhoven, The Netherlands) spectroscopy was performed using an energy dispersive spectrum to identify elementary compositions of the Eh-AgNPs. Zeta potential of the Eh-AgNPs was calculated usinga zeta potential analyser (Zetasizer, ver. 7.11, Malvern Instruments Ltd., Malvern, UK).

### 2.4. Antimicrobial Activity

The disk-diffusion method [33] following Clinical and Laboratory Standards Institute (CLSI) guidelines [34] was applied to assess the antibacterial efficacy of the Eh-AgNPs against pathogenic Gram-positive *Bacillus cereus* (ATCC 10876) and *Staphylococcus aureus* subsp. *aureus* (ATCC 11632); Gram-negative *Escherichia coli* (ATCC 10536) and *Pseudomonas aeruginosa* (ATCC 10145); and MDR strains of *Escherichia coli* (NCTC 13351), *Enterococcus faecium* (ATCC 700221), *Staphylococcus aureus* subsp. *aureus* (ATCC 33592), and *Streptococcus pneumoniae* (ATCC700677), which were purchased from Bio-Focus Saintifik Sdn Bhd, Malaysia. Antibiotic disks (BD BBL Sensi-Disc, Beckton Dickinson, Franklin Lakes, NJ, USA) of ampicillin (10 μg) and ciprofloxacin (5 μg) were used for the Gram-positive and Gram-negative bacteria, respectively. Antifungal activity of the Eh-AgNPs against *C. albicans* (ATCC 10231) was assessed using the disk-diffusion method [33] following CLSI guidelines [35], with the antifungal itraconazole (10 µg, Tokyo Chemical Industry, Tokyo, Japan) used as the positive control.

The pathogenic and MDR bacteria were grown on nutrient broth (HiMedia Lab. Ltd., Mumbai, India) at 37 °C for 24 h while pathogenic fungal strains were grown on potato dextrose broth (HiMedia Lab. Ltd., Mumbai, India) at 30 °C for 24 h in sterile screw-cap test tubes. Thereafter, the pathogenic and MDR microbes were cultured on nutrient agar media (HiMedia Lab. Ltd., Mumbai, India) at 37 °C for 24 h while fungal isolates were cultured on potato dextrose agar media (HiMedia Lab. Ltd., Mumbai, India) at 30 °C for 24 h in Petri plates. Colony suspension (~10^6^ CFU/mL) for each microorganism was maintained at an equivalent to 0.5 McFarland standard prepared in Mueller-Hinton agar (MHA) (HiMedia Lab. Ltd., Mumbai, India) at pH 7. Microorganisms were swabbed on Petri plates containing MHA to perform the antimicrobial susceptibility test following the disk-diffusion method [33]. Sterile Whatman No. 1 filter paper (Sigma-Aldrich, St. Louis, MO, USA) disks of 6 mm diameter were impregnated with 6 µg and 10 µg of Eh-AgNPs to test the antimicrobial activity against the pathogenic microbes and MDR bacteria, respectively. The Eh-AgNP disks and/or antibiotic disks were placed in the MHA and incubated for 24 h at 37 °C and 30 °C for bacterial and fungal isolates, respectively. A vernier calliper (Mitutoyo, S-530, Mitutoyo Co., Kawasaki, Japan) was used to measure the zone of inhibition (ZOI). Simultaneously, similar experiments were conducted using freshly prepared CFE and AgNO_3_ solution as the negative control.

Minimum inhibitory concentration (MIC) was estimated by the broth macrodilution method [36] following CLSI guidelines [37]. For this reason, a trial-and- error method was applied to standardize the working concentrations of Eh-AgNPs [38]. Different concentrations of the Eh-AgNPs in 4 mL nutrient broth were prepared in test tubes following two-fold serial dilution. An aliquot (0.2 mL) of microbial suspension (~10^6^ CFU/mL) was added to each test tube. A positive control with microbial suspension (~10^6^ CFU/mL) in nutrient broth and negative control with nutrient broth were also maintained. Thereafter, the test tubes with pathogenic and MDR bacteria were incubated at 37 °C for 24 h while those of the pathogenic fungus were incubated at 30 °C for 24 h. The lowest concentration of the Eh-AgNPs at which there was no visible growth of the organisms was considered to be the MIC [36].

### 2.5. Statistical Analysis

The results of the triplicate experiments were expressed as mean ± SD. One-way ANOVA followed by Tukey’s honestly significant difference (HSD) test with a significance level of 0.05 were analysed using IBM SPSS for Windows, Version 22.0 (IBM Corp., Armonk, NY, USA).

## 3. Results

### 3.1. Synthesis and Characterization of Eh-AgNPs

**Figure 1:**
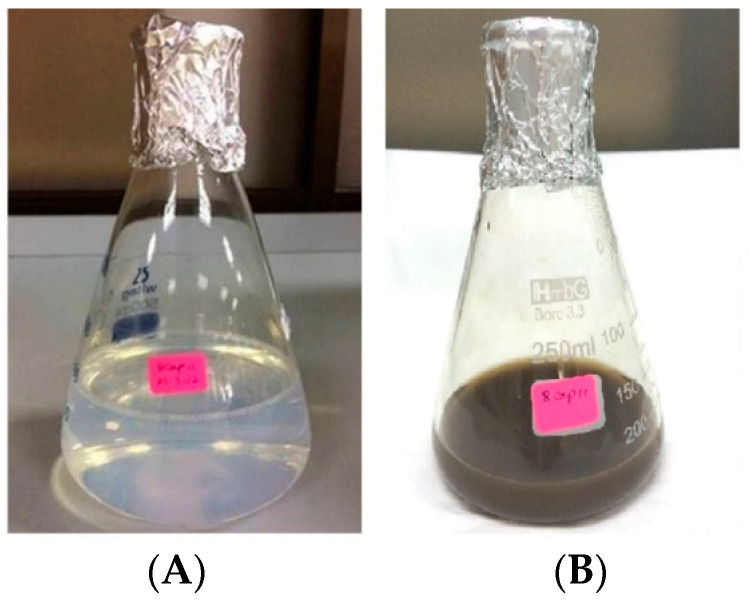
Visible colour change of the reaction mixture before (**A**) and after (**B**) sunlight exposure.

**Figure 2:**
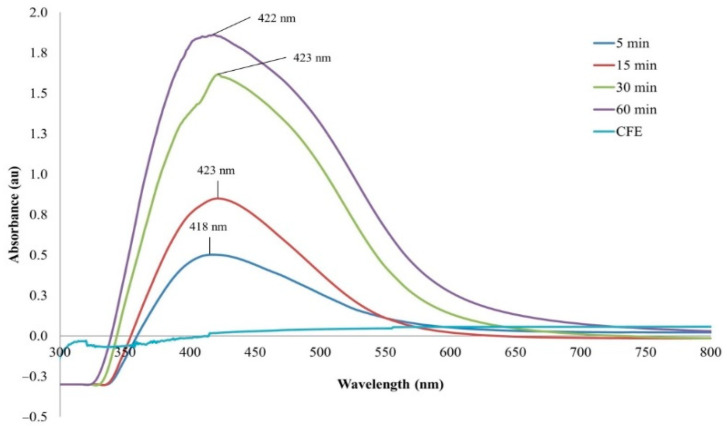
UV-Vis spectrum of Eh-AgNPs.

**Figure 3:**
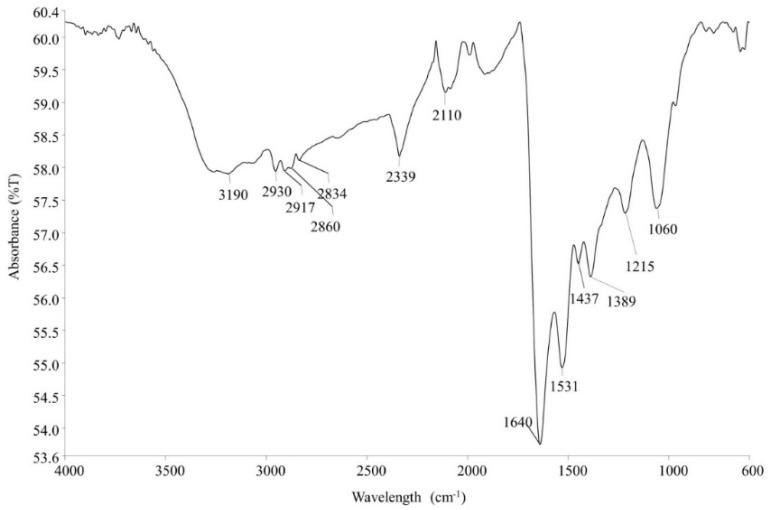
Fourier-transform infrared (FTIR) spectrum of Eh-AgNPs.

**Figure 4:**
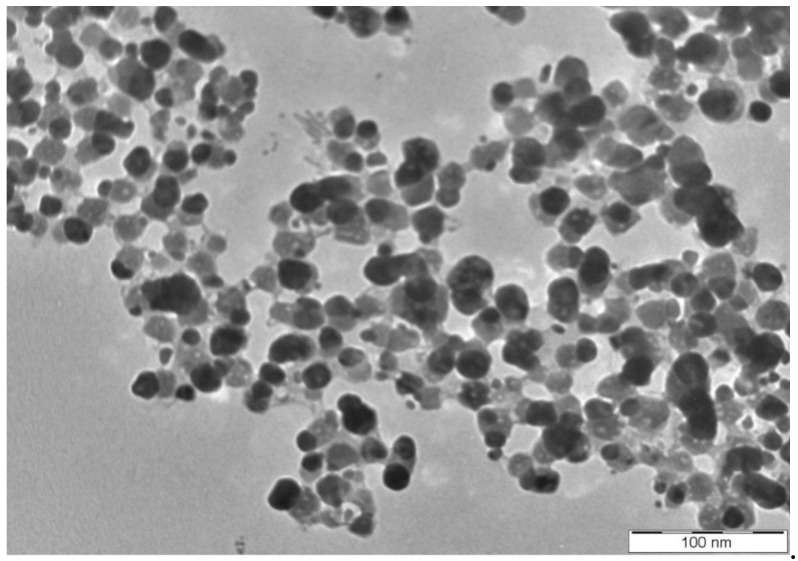
TEM image of Eh-AgNPs.

**Figure 5:**
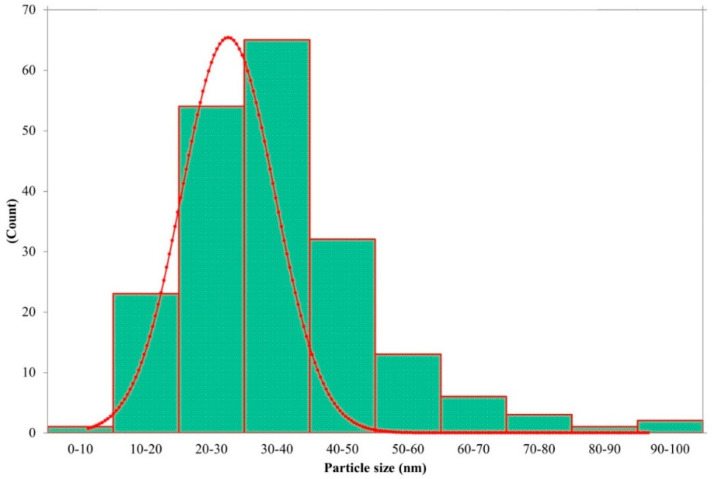
Histogram of Eh-AgNP size distribution.

**Figure 6:**
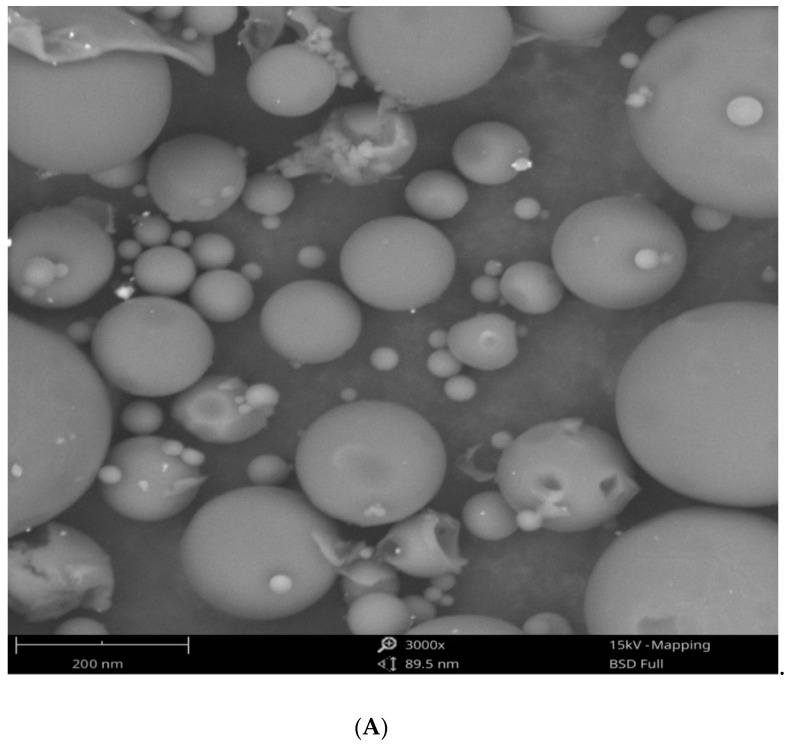

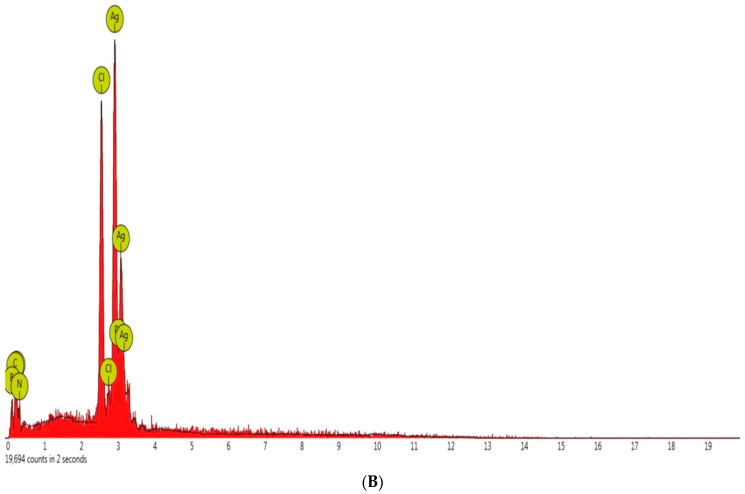
SEM image (**A**) and EDX image (**B**) of Eh-AgNPs.

**Figure 7:**
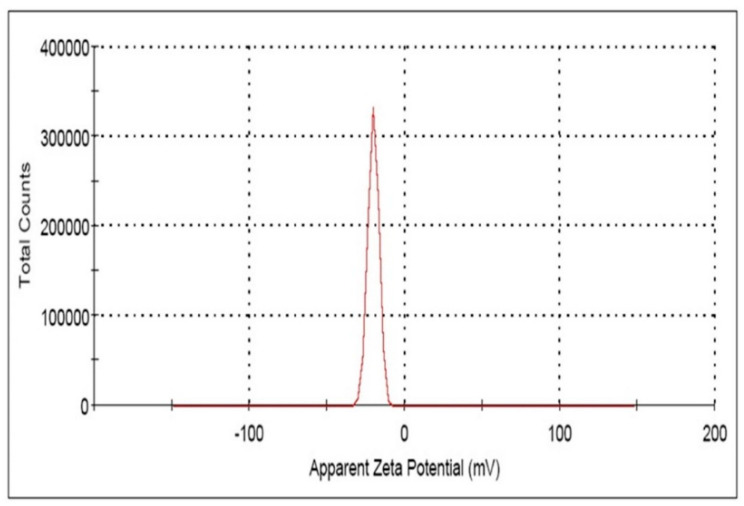
Zeta potential spectrum of Eh-AgNPs.

The reaction mixture of the CFE and AgNO_3_ solution turned from whitish to deep yellowish brown (Figure 1) within 60 min. Formation of Eh-AgNPs was confirmed by UV-Vis spectrum of the reaction mixture. A clear surface plasmon resonance (SPR) was formed within 5 min resulting in 418 nm of wavelength in the UV-Vis spectrum (Figure 2). In contrast, little colour change was observed in the reaction mixture kept in the dark while the negative control mixture revealed no colour change. FTIR analysis was carried out to assess possible functional biomolecules responsible for the reduction of Ag^+^ to Ag^0^. The FTIR spectrum of the Eh-AgNPs showed a number of absorption peaks (Figure 3). The absorbance peaks at 3190 cm^−1^ and 2834 cm^−1^ corresponded to N-H functional groups of primary amine in proteins, while the peak at 1531 cm^−1^ corresponded to C=N stretching vibration due to secondary amide in proteins [39]. The peaks at 2930, 2917, and 2860 cm^−1^ were attributed to C-H stretching of aliphatic groups [39]. The peak at 2339 cm^−1^ corresponded to asymmetric O=C=O stretching of CO_2_, while the weak band at 2110 cm^−1^ was due to C≡C stretching vibration of the aliphatic groups [40]. The band at 1640 cm^−1^ attributed to primary amide was due to C=O stretching in proteins and H-O-H deformation of water [39]. The peaks at 1389, 1215, and 1060 cm^−1^ were assigned to the symmetric deformation of the vibration of O-H groups in alcohols, C-O stretching of aromatic ether, and S=O stretching vibration of sulfoxides, respectively [40]. The peaks at around 808 cm^−1^ and below were attributed to C–H aromatic vibrations [41]. The Eh-AgNPs were mostly spherical, polydispersed, and inconsistent in size as shown in the TEM image (Figure 4). Descriptive analysis and a particle-size distribution histogram of 200 arbitrarily selected nanoparticles were obtained from TEM microgram. The particle-size histogram (Figure 5) revealed the size of the Eh-AgNPs to be in the range from 9.91 nm to 92.54 nm, with a mean of 35.13 ± 14.24 nm. Surface morphology of the Eh-AgNPs as revealed from the SEM image (Figure 6A) showed the formation of nanoparticles with a higher degree of accumulation without any impurities. The presence of silver was confirmed through EDX. The EDX spectrum (Figure 6B) indicated a strong signal at 3 keV with different elemental ratios of Ag in different parts. A higher mass percentage of Ag atoms (60.91%) was observed in comparison to atoms of chlorine (11.27%), boron (15.42%), carbon (8.53%), and nitrogen (3.87). The findings of SEM-EDX analysis (Figure 6A,B) showed the metallic nature of the synthesized AgNPs. Zeta potential was carried out to measure the magnitude of the electrostatic or charge repulsion or attraction among the nanoparticles and to determine the stability of the Eh-AgNPs. The mean of the zeta potential values was found as −19.73 ± 3.94 mV with the range of −20.3 ± 3.53 to −19.3 ± 3.97 mV (Table 1, Figure 7).

### 3.2. Antimicrobial Activity

**Figure 8:**
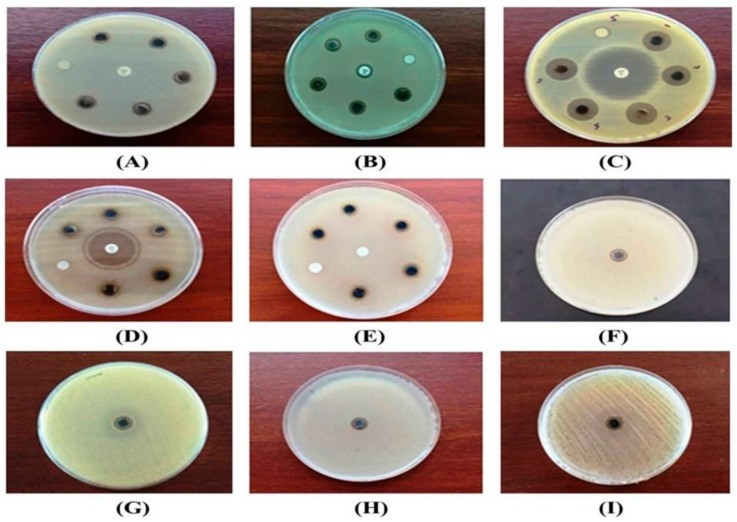
Antimicrobial activity of Eh-AgNPs against (**A**) *B*. *cereus* (ATCC 10876), (**B**) *S. aureus* subsp. *aureus* (ATCC 11632), (**C**) *E. coli* (ATCC 10536), (**D**) *P*. *aeruginosa* (ATCC 10145), (**E**) *C. albicans* (ATCC 10231), (**F**) MDR *S*. *pneumoniae* (ATCC 700677), (**G)** MDR *E. faecium* (ATCC 700221), (**H**) MDR *S*. *aureus* subsp. *aureus* (ATCC 33592), and (**I**) MDR *E*. *coli* (NCTC 13351).

The results of the antimicrobial study of the activity of Eh-AgNPs against the experimental pathogenic bacteria are presented in Table 2 and Figure 8A–D. A significant difference (*p* < 0.05) was observed among the ZOIs of different pathogenic microbes. The Eh-AgNPs at 6 μg concentration resulted in the highest ZOI against *E*. *coli* (ATCC 10536), of 15.16 ± 0.05 mm with a MIC value of 1.25 μg/mL, while the lowest ZOI was observed against *P*. *aeruginosa* (ATCC 10145) as 7.18 ± 0.04 mm with a MIC value of 2.25 μg/mL. The ZOIs of 30.48 ± 0.08 mm and 30.10 ± 0.07 mm produced by the Eh-AgNPs against *E*. *coli* (ATCC 10536) and *P*. *aeruginosa* (ATCC 10145), respectively, were found to be lower than that of the positive control, ciprofloxacin. By contrast, the ZOI of 11.20 ± 0.07 mm produced by the Eh-AgNPs against *S*. *aureus* subsp. *aureus* (ATCC 11632) was higher than the ZOI of 10.14 ± 0.05 mm produced by the positive control, ampicillin. In contrast, the Eh-AgNPs exhibited better antimicrobial activity against *B*. *cereus* (ATCC 10876), producing a ZOI of 9.14 ± 0.05 mm, while that strain was found to be resistant against the positive control, ampicillin. The CFE and AgNO_3_ produced no inhibitory activity against the experimental microbes. The fungal strains of *C*. *albicans* (ATCC 10231) were found to be resistant against the conventional antibiotic, itraconazole—10 μg, whereas the synthesized Eh-AgNPs effectively inhibited their growth, displaying an 8.24 mm ZOI with MIC of 2.0 μg/mL (Table 2, Figure 8E). The Eh-AgNPs at 10 μg concentration resulted in a significant difference (*p* < 0.05) in the ZOIs among different MDR bacteria (Table 2, Figure 8F–I). The highest ZOI was observed with MDR *E. faecium* (ATCC 700221), at 13.20 ± 0.07 mm, with a MIC value of 2.00 μg/mL, while the lowest ZOI was observed with MDR *S*. *pneumoniae* (ATCC 700677) at 10.98 ± 0.08 mm, with a MIC value of 6.00 μg/mL.

## 4. Discussion

### 4.1. Synthesis and Characterization of Eh-AgNPs

Synthesis of Eh-AgNPs was rapid (5 min), exhibiting a change in colour of the CFE and AgNO_3_ solution due to excitation of strong plasmon resonance resulting from oscillation of silver ions and SPR in the visible region [41], which might correspond to the synthesis of spherical Eh-AgNPs [20,42,43]. The Eh-AgNPs formed at 15, 30, and 60 min time intervals were found to be larger and aggregated in size. The reaction mixture kept in the dark produced some colour change, indicating that both sunlight exposure and CFE are required for the synthesis of Eh-AgNPs. The study clearly demonstrated that the supernatant of the experimental endophytic *E*. *hormaechei* possesses reducing and capping agents [15,16,17]. Bacteria-mediated synthesis of AgNPs follows a bottom-up approach. Although the exact mechanism of bacteria-mediated extracellular synthesis of AgNPs is not known, several hypotheses outlining the role of bacterial cell biomolecules involved in the synthesis process have been proposed [9].

The Eh-AgNPs in the FTIR spectrum analysis revealed the presence of proteins and alcohols, among other components. These findings demonstrated the interaction of bacterial cell-secreted functional biomolecules such as amine, amide, and hydroxyl groups with the surfaces of Eh-AgNPs, where these groups acted as capping areas for the stability of the nanoparticles as well as reducing and stabilizing agents that enabled the production of Eh-AgNPs from metal salts [9,12,14,16]. However, the presence of secondary metabolites observed in the FTIR study might be a result of horizontal gene transfer (HGT) to the endophytic *E*. *hormaechei* from the host plant [44].

Bacteria-mediated AgNPs may be of variable shapes such as spherical, quasi-spherical, cuboidal, disk-shaped, triangular, hexagonal, rod-shaped, irregular, etc. with the size ranging from 0.5 to 595.00 nm [9,15,16]. The present study corroborated some earlier studies reporting size inconsistency among spherical and polydispersed nanoparticles synthesized using various microbes [28,42,43]. However, the size and shape of extracellularly produced AgNPs depend upon the culture media, reducing agent, and bacterial species used during the synthesis [9].

The strong signal at 3 keV in the EDX spectrum (Figure 6B) was due to SPR, confirming the presence of silver [28]. Like the present study, different ratios of elemental composition in the synthesized nanoparticles were reported earlier in the literature [28,42]. The presence of other elements such as Cl, B, C, and N (Figure 6B) observed in the EDX spectrum might be due to the CFE and the carbon grid that were used during sample preparation [28,42].

Zeta potential (ζ) refers to the electro-kinetic potential in colloidal systems, which is related to the short- or long-term stability of emulsions. Emulsions with high negative or positive ζ are electrically stabilized while emulsions with low ζ tend to coagulate or flocculate. The larger the zeta potential, the greater the repulsive force; and the more stable the AgNP, the less the tendency of the suspension system to move towards aggregation [42,45]. The Zeta potential values in the present study (Table 1) indicated that Eh-AgNPs are able to form a moderately stable colloid in aqueous suspension [42,45]. The synthesized nanoparticles were found to be stable after more than 6 months. The present study clearly confirmed the higher stability of nanoparticles compared to those synthesized from *Streptomyces xinghaiensis* OF1 strain (−15.7 mV) [43], *Bacillus* sp. MB353 (−18.36 mV) [41], and *Bacillus cereus* A1-5 (−17.5 mV) [42].

### 4.2. Antimicrobial Activity

Interpretive standards (susceptible, intermediate, or resistant), as measured by the ZOI diameter and MIC for different microbes, are species-dependent [46]. Hence, the antimicrobial susceptibility of the Eh-AgNPs was compared to that of the conventional antibiotics used in this study. The Eh-AgNPs in the present study exhibited the highest antimicrobial activity against Gram-negative *E*. *coli* (ATCC 10536) followed by Gram-positive *S*. *aureus* subsp. *aureus* (ATCC 11632), *B*. *cereus* (ATCC 10876), and Gram-negative *P*. *aeruginosa* (ATCC 10145), with mean ZOIs of 15.15 ± 0.05, 11.20 ± 0.07, 9.14 ± 0.05, and 7.18 ± 0.04 mm, respectively (Table 2). In a recent study, endophytic *Pseudomonas* sp. ef1-mediated spherical AgNPs (20–70 nm in size) at a concentration of 25 μL were reported to exhibit the highest antimicrobial activity against Gram-negative *E*. *coli* followed by *K. pneumoniae* with ZOIs of 17 and 16 mm, respectively, while Gram-positive *S. aureus* and Gram-negative *Acinetobacter baumannii* and *Citrobacter koseri* exhibited the same ZOI of 15 mm [28]. Endophytic *Aneurinibacillus migulanus* 141-mediated AgNPs (20–60 nm in size) at a concentration of 50 μL (10 μg/μL) were reported to display the highest antibacterial activity against Gram-negative *P. aeruginosa* (MTCC 7903) followed by Gram-positive *B. subtilis* (MTCC 121), *Gram-negative E. coli* (MTCC 7410), *K. pneumoniae* (MTCC 7407), and Gram-positive *S. aureus* (MTCC 7443) with ZOIs of 21, 19, 18, 17, and 16 mm, respectively [29]. *Bacillus thuringiensis*-mediated AgNPs (42 ± 7 nm mean size) at a concentration of 50 μL (50 μg/mL) were reported to show maximum antimicrobial activity against Gram-negative *E*. *coli* O157H7 followed by Gram-positive *S*. *aureus* (PTCC 1112), Gram-negative *K*. *pneumoniae* (PTCC 1053) and *P*. *aeruginosa* (PTCC 1310), and Gram-positive *Listeria monocytogenes* (PTCC 1298) and *E*. *faecalis* (PTCC 1237) with ZOIs of 19.7 ± 0.58, 18.7 ± 1.5, 18.0 ± 1.15, 17.7 ± 0.58, 17.3 ± 0.58, and 16.2 ± 0.29 mm, respectively, while the MIC was within the range of 6.25–12.5 μg/mL [47]. Another study of antimicrobial potential reported that endophytic *Pseudomonas fluorescens* CA 417-mediated AgNPs (5–50 nm size) at a concentration of 50 μL (10 mg/mL) were more effective against Gram-positive *B. subtilis* (MTCC 121) followed by Gram-negative *E. coli* (MTCC 7410), Gram-positive *S. aureus* (MTCC 7443), Gram-negative *P. aeruginosa* (MTCC 7903), and *K. pneumoniae* (MTCC 7407) with mean ZOIs of 12.33 ± 0.57, 11.66 ± 0.57, 11.00 ± 1.00, 9.66 ± 0.57, and 8.00 ± 1.00 mm, respectively [30]. Similarly, endophytic bacterium EH 419-mediated AgNPs (10–60 nm size) at a concentration of 500 μg were reported to possess the highest antibacterial activity against Gram-negative *P*. *aeruginosa* (MTCC 7903) followed by Gram-negative *E*. *coli* (MTCC 7410); Gram-positive *S*. *aureus* (MTCC 7443) and *B*. *subtilis* (MTCC 121); and Gram-negative *K*. *pneumoniae* (MTCC 7407) with ZOIs of 20, 15, 14, 13, and 11 mm, respectively [31]. Therefore, the findings of the present study are in close agreement with earlier findings reported in literature [28,29,30,31,47].

Among human microbiota, *C*. *albicans* is the most predominant fungal species that causes a wide range of infections. It is estimated that infections resulting from *Candida* sp. cause direct medical costs totalling $3 billion in the USA [1]. Recent reports of *Candida* sp. resistance to antifungal drugs are a serious concern in the healthcare setting [48]. Earlier reports in the literature suggest that bacteria-mediated AgNPs can be an alternative, safe, and effective measure to treat *C*. *albicans* [28,43,49,50]. The Eh-AgNPs in the present study displayed more effective antifungal activity than the conventional antibiotic, itraconazole. Similarly, *Bacillus methylotrophicus* DC3-mediated AgNPs (7–31 nm size) at a concentration of 3 μg were reported to be sensitive antimicrobial agents against *C*. *albicans* while it was resistant to the conventional antibiotic, cycloheximide-3 μg [50]. However, higher antifungal efficacy with a 15-mm ZOI was reported with *Pseudomonas* sp. ef1-mediated AgNPs (20–70 nm size, 25 μL conc.) against *C. albicans* [28]. Additionally, the effectiveness of *Bacillus safensis* LAU 13-mediated AgNPs (5–95 nm size) against *C. albicans* was reported with a MIC of 40 μg/mL [49]. Furthermore, in the present study, the Eh-AgNPs exhibited a lower MIC value than did the *Streptomyces xinghaiensis* OF1-mediated AgNPs (5–20 nm size) against *C. albicans* (ATCC 10231), with a MIC of 32 μg/mL [43]. The variations in the MIC values may be attributed to differences in the type of strain used, methods of evaluation [51], and the concentrations of AgNPs used [52]. Although the exact mode of antifungal action of AgNPs against *Candida* sp. is not yet known, several mechanisms have been elucidated in the literature [53,54,55,56].

The present study revealed that Eh-AgNPs possess antibacterial potential against various MDR pathogens (Table 2, Figure 8F–I). Some previous studies have also reported antimicrobial efficacy of different bacteria-mediated AgNPs against Gram-positive and Gram-negative drug-resistant microbes [23,57,58,59,60]. The bactericidal efficacy of AgNPs synthesized using a silver-tolerant bacteria, *Bacillus cereus*, against ESKAPE pathogens such as *Enterococcus faecium* (MCC 2763), methicillin–resistant *Staphylococcus aureus* (ATCC 33591, MTCC 1430), *Klebsiella pneumoniae* (ATCC 35657, MTCC 432), *Acinetobacter baumannii* (ATCC 19606, MTCC 1920), *Pseudomonas aeruginosa* (ATCC 27853, MTCC 1688), *Enterobacter aerogenes* (MTCC 111), and *Enterobacter* sp. (MCC 2296) was reported by Khan and co-workers [58]. The authors reported that the synthesized spherical AgNPs (45–140 nm size, conc. 7.81–500 μg/mL) exhibited bactericidal efficacy in the range of 15.62–250 μg/mL and that *P. aeruginosa* (ATCC 27853) was more susceptible in comparison to *K. pneumoniae* (MTCC 432) or MRSA (ATCC 33591) [58]. Inhibition activity of *Streptomyces* sp. Al-Dhabi-89 mediated AgNPs was reported to exhibit the lowest MIC value of 7.81 μg/mL against drug-resistant *Escherichia coli* (ESBL 4345), *Acinetobacter baumannii* (MDR 4273), *Acinetobacter baumannii* (MDR 7077), MDR *Staphylococcus aureus* (WC 25 V 880854), and MDR *Staphylococcus aureus* (V 552) followed by drug-resistant *Acinetobacter baumannii* (MDR 4474), *Acinetobacter baumannii* (4414), *Acinetobacter baumannii* (MRO 3964), *Proteus mirabilis* (DR 4753), *Staphylococcus aureus* (ATCC 43300), and *Staphylococcus aureus* (TC 7692) with MIC value of 15.6 μg/mL [57]. The MIC value of the AgNPs was reported as 31.25 μg/mL against drug-resistant *A. baumannii* (MDR 4414), *Escherichia coli* (ATCC 35218), and *Pseudomonas aeroginosa* (MDR 4406), while drug-resistant *Enterococcus faecium* (VRETC 773) and *Enterococcus faecium* (VRE UR 83198) displayed the highest MIC value as 62.5 μg/mL [57]. Our previous study with endophytic *Pantoea ananatis*-mediated AgNPs at 10 μg concentration displayed 10.16, 10.20, and 12.16 mm ZOIs against MDR strains of *S*. *aureus* subsp. *aureus* (ATCC 33592), *S*. *pneumoniae* (ATCC700677), and *E*. *faecium* (ATCC 700221), respectively [23]; these were lower than the present findings shown in Table 2. The antimicrobial activity of *Bacillus brevis* (NCIM 2533)-mediated AgNPs (41–68 nm size) at different concentrations of 5, 10, 15, and 20 μL was studied against MDR clinical isolates of Gram-positive *Staphylococcus aureus* and Gram-negative *Salmonella typhi* [59]. AgNPs were reported to exhibit maximum antimicrobial activity against MDR *S*. *aureus* with mean ZOIs of 14, 15, 16, and 19 mm, respectively, while moderate antibacterial activity was reported against MDR *S*. *typhi* with mean ZOIs of 0, 7, 7 and 7.5 mm at 5, 10, 15, and 20 μL concentrations, respectively [59]. Another study with *Acinetobacter baumannii* mediated AgNPs having less stable (zeta potential of −11.7 mV) and a higher particle-size range (37–168 nm) than the AgNPs in the present study were reported to exhibit inhibitory effects against several Gram-negative MDR pathogens, such as *Escherichia* *coli* (E3), *Pseudomonas* *aeruginosa* (P21), and *Klebsiella* *pneumoniae* (K32), with a MIC of 1.53–3.125 μg/mL [60]. AgNPs synthesized using *A*. *baumannii* were tested against β-lactams-, aminoglycosides-, and quinolones-resistant *E*. *coli*, *P*. *aeruginosa*, and *K*. *pneumoniae*, and the results showed that the nanoparticles were effective against those microbes with MIC values of 3.1, 1.56, and 3.1 μg/mL, respectively [60]. However, a concrete comparison with other study reports describing different levels of ZOIs and MICs is difficult due to the differences in procedures and concentrations used [29,42,57,59,61] and biological strains used [28,41,42,47,61]. Besides, the shape and size of the synthesized AgNPs may affect their antimicrobial activity against both Gram-positive and Gram-negative bacteria and fungi [62]. The antimicrobial activity of AgNPs against pathogenic microbes may be subject to variation due to differences in microbial structure, molecular dynamics, and sequence [63]. Furthermore, inter-strain differences in bacteria may be attributable to genome versatility resulting from heterogeneity in the bacterial accessory genome, i.e., its lysogen [64], and thus account for the observed antimicrobial variability.

Although the exact mechanism of AgNP antimicrobial activity is yet to be known, several hypotheses have been described to illustrate the antimicrobial mechanisms of AgNPs in the literature [10,13,14]. In brief, (i) adhesion of AgNPs to the microbial cell wall causes disintegration of the cell wall and membrane, leading to leakage of intracellular content and finally disruption of cell integrity and cell death; (ii) intracellular penetration of AgNPs causes degradation and denaturation of bacterial deoxyribonucleic acid (DNA) with ribosomal denaturation, leading to the inhibition of translation and protein synthesis, the inhibition of sugar metabolism resulting from inactivation of phosphomannose isomerase, and the inhibition of protein biosynthesis resulting from inactivation of the enzymatic protein tryptophanase (TNase); (iii) AgNPs produce free radicals and reactive oxygen species (ROS), leading to increased oxidative stress followed by cytotoxic and genotoxic effects; and (iv) AgNPs modulate signal transduction in microbial cells through dephosphorylation of tyrosine, leading to bacterial growth inhibition. However, more research at the molecular level will be necessary to conclusively identify the mechanism of antimicrobial action of AgNPs.

## 5. Conclusions

This study provided insights into the emerging role of endophytes towards the synthesis of nanoparticles. To the best of our knowledge, this is the first report on biosynthesis of AgNPs using endophytic *E. hormaechei*. The synthesis took place within 5 min, which was a rapid process. The Eh-AgNPs were spherical in shape and displayed significant antimicrobial activity against pathogenic *B. cereus* (ATCC 10876), *S. aureus* subsp. *aureus* (ATCC 11632), and *C. albicans* (ATCC 10231) compared to conventional antibiotics. Additionally, the Eh-AgNPs showed moderate antibacterial activity against MDR strains of *S. pneumoniae* (ATCC700677), *E. faecium* (ATCC 700221), *S. aureus* subsp. *aureus* (ATCC33592), and *E. coli* (NCTC 13351). It is evident from the present study that endophytic *E. hormaechei* could be a novel candidate in facilitating rapid and eco-friendly biosynthesis of AgNPs with potential applications aimed at tackling the antibacterial resistance problem worldwide. Further studies concerning downstream processes, biological and environmental toxicity, as well as mechanisms of antimicrobial action at the molecular level will be required for the application of Eh-AgNPs to the development of independent or synergistic antimicrobial agents for AMR management. However, the present study extends the frontiers of biotechnological application for endophytic bacteria in the field of nanotechnology. The results of the present investigation are promising and feature a growing scientific breakthrough in unexplored roles for endophytic microbes in the formulation of new and alternative antimicrobial agents for the control of drug-resistant pathogens in the near future.

## Figures and Tables

**Table 1:** Zeta potential value of AgNPs synthesized by *E*. *hormaechei*.

Triplicate Study	Zeta Potential (ζ) (mV)	Area (%)	Conductivity (mS/cm)
	Mean ± SD (mV)		
1.	−19.3 ± 3.97	100	0.0362
2.	−19.6 ± 4.32	100	0.0101
3.	−20.3 ± 3.53	100	0.00980

**Table 2:** Antimicrobial activity of the synthesized AgNPs against pathogenic and MDR microbes.

Microbes	ZOI (mm)	MIC (μg/mL)	Control
	Pathogenic Microbes ^1^		
*B*. *cereus* (ATCC 10876)	9.14 ± 0.05 ^c^	1.75	Ampicillin (10 μg): resistant
*S*. *aureus* subsp. *aureus* (ATCC 11632)	11.20 ± 0.07 ^b^	1.50	Ampicillin (10 µg): 10.14 ± 0.05
*E*. *coli* (ATCC 10536)	15.16 ± 0.05 ^a^	1.25	Ciprofloxacin (5 µg): 30.48 ± 0.08
*P*. *aeruginosa* (ATCC 10145)	7.18 ± 0.04 ^e^	2.25	Ciprofloxacin (5 µg): 30.10 ± 0.07
*C*. *albicans* (ATCC 10231)	8.24 ± 0.05 ^d^	2.00	Itraconazole (10 µg): resistant
	MDR bacteria ^2^		
*S*. *pneumoniae* (ATCC 700677)	11.24 ± 0.05 ^C^	5.25	-
*E*. *faecium* (ATCC 700221)	13.20 ± 0.07 ^A^	2.00	-
*S*. *aureus* subsp. *aureus* (ATCC 33592)	10.98 ± 0.08 ^D^	6.00	-
*E*. *coli* (NCTC 13351)	12.24 ± 0.05 ^B^	3.75	-

^1^ AgNP concentration: 6 μg/disc. ^2^ AgNP concentration: 10 μg/disc. Results are observed as mean ± SD. Significant differences among the mean values of the pathogenic microbes and of MDR bacteria determined by the Tukey’s HSD test (*p* < 0.05) are indicated by different letters (^a–e^ and ^A–D^, respectively).

## Data Availability

The data presented in this study are available in this article.
